# Current Hospital Topics

**Published:** 1906-12-01

**Authors:** 


### HOSPITAL ADMINISTRATION.

### CONSTRUCTION AND ECONOMICS.

### CURRENT HOSPITAL TOPICS.

#### The Hospital Funds in Manchester.

Heretofore the Sunday and Saturday Funds at Manchester have been worked under one committee. It has now been determined that the Sunday and Saturday Funds shall be separated, so that each Fund may have the advantage of distinct and vigorous organisations. The results of each year's collections by the two Funds are however to be pooled. They will then be distributed amongst the various charities under a code of regulations for the guidance of the Joint Committee of Distribution charged with this duty.

#### Addenbrooke's Hospital, Cambridge.

Very strenuous efforts have been made during recent years to secure the utmost economy in all departments of this hospital. Each quarter the General Committee issue a statement showing the expenditure under various heads. That for the quarter ended September 30 last shows a reduction of upwards of £65 in the ordinary expenditure as compared with the previous year, savings having been effected in provisions, surgery and dispensary, and domestic expenses. When due allowance has been made for the difference in the number of patients under treatment, and generally, an examination of the figures has led us to the conclusion, that, the whole of the officers must be working well under Mr. Albert Pell's chairmanship, and that these reductions represent an increase in efficiency. We have noticed the same tendency of late in previous quarterly reports. We draw attention to these facts in the hope that the recent meeting at the Guildhall, Cambridge, may result in considerably increasing the resources of this most useful and much needed hospital. It deserves the warm support of all intelligent residents in the town and county of Cambridge.

#### North Devon Infirmary, Barnstaple.

In consequence of a resolution adopted by the governors last May, owing to the unhealthiness of the existing buildings, where four nurses and several patients contracted typhoid fever during the previous six months, extensive structural alterations have been made to the old buildings which were put up in 1825. The reconstruction includes the erection of two sanitary towers, one to each wing, containing bathrooms and lavatories, with the addition of a small isolation ward having a bathroom, on each floor. An attempt has been made to improve the ventilation and sanitation of the old wards by the addition of several new windows and the heating arrangements in the casualty department have been reconstructed. Towards the cost of this reconstruction Lord Fortescue has collected £1,200, £1,530 has been received from the proceeds of a bazaar held in November, and a legacy of £1,000 has brought the total amount available for the Building Fund up to £3,730. It it hoped when this reconstruction has been completed that the Barnstaple and North Devon Infirmary will be found in every way more convenient, sanitary and up to date as a building for the reception of patients suffering from acute disease.

#### A Hospital Monthly Magazine.

In our issue of the 3rd ulto. we drew attention to a practice which is increasing in the colonies, of issuing a small paper once a month, to contain a full account of the most interesting items in the work of the particular hospital whose cause it advocates. Then these papers obtain a circulation not only amongst the patients and staff but amongst the governors also. We are reminded by the editor that Alderman Sparke has for thirteen years issued *The Leeds Hospital Magazine*, published at ½d., being a record of the Leeds Workpeople's Hospital Fund, of which he sends us a copy. It consists of sixteen pages, including the cover, and would seem from its contents to be mainly confined to promoting the Leeds Workpeople's Hospital Fund. A page is devoted to the Hospital Work and Attendances of the Leeds charities, and the rest of the space is almost entirely occupied by a short story, columns devoted to "Light Bits," "Useful Bits," short reports of different meetings and advertisements. We confess to the feeling that if the active co-operation of the Leeds Infirmary and the other local medical charities were to be secured, it should be possible to provide more suitable copy of undoubted interest which could not fail to help the finances and increase the public interest in the Leeds Medical Charities.

#### A Canadian Philanthropist.

Mr. John Ross Robertson has had an interesting career in journalism, having at the age of sixteen started and edited a paper for boys, which he printed and published himself. He has continued in journalism all his life, and is now the publisher of one of the big Canadian evening dailies, the *Evening Telegram*. Having amassed a fortune he has thrown himself actively into hospital work, and is at present chairman of the Board of Trustees of the Sick Children's Hospital, Toronto, to which institution he has devoted much time and money. His latest gift is a new nurses' residence which is situated between La Plante Avenue and Elizabeth Street, Toronto. Mr. Robertson has defrayed the entire expenses of this imposing building, including furniture and equipment, at a cost of about 120,000 dollars. It is one of the glories of the English nation that its palaces of pain are supported by voluntary contributions. Mr. Robertson's splendid generosity cannot fail to set an example to many other wealthy men in Canada, and so is likely to prove of value to the voluntary hospitals there. We congratulate Mr. Robertson upon the evidence which the plan affords of the infinite care, the ability and brains which have been brought to bear upon the planning and design of the nurses' residence, which seems likely to prove, when opened, one of the most complete and up-to-date buildings of the kind in the world.

### A City Hospital Conference.

The New York Committee has lost no time but has done some practical work at the commencement of the winter session. At a meeting held on October 30 the members of the Hospital Conference decided to study systematically various subjects relating to hospital management. These include hospital expenditure, uniform accounts, state inspection and municipal aid, the distribution and classification of hospitals and hospital beds in relation to the needs of the community, ambulance service, dispensaries, medical organisation and medical education, paying patients, the co-operation of hospitals with each other and with relief agencies, and the treatment of patients in their own homes. The method to be pursued is novel, but practical. A committee of investigation consisting of ten members was appointed, which was then sub-divided into committees of one, to each of whom will be assigned one or more of the subjects just enumerated for investigation and report. The procedure to be followed is, that this committee is to have the right to offer reports, and to make recommendations to the conference as a body. Each of its members has the right to present to the conference at any regular meeting a report on the subject especially assigned to him, and the reading of such reports will constitute part of the regular order of business of each conference. No doubt with the view of stimulating individual effort each member of the committee is granted the right to publish his own reports as personal documents. On the other hand it is the intention to publish as official documents of the conference those reports which may be adopted by it, providing their publication is ordered by the conference on the recommendation of the executive committee. We hope that some active spirits in the English hospital world may decide to organise similar hospital conferences in the greater cities throughout the country. They must be attended with considerable benefit to all the interests concerned by disseminating accurate information, and exciting much interest locally. More important still they should tend to prepare the way for the co-ordination and co-operation of all the voluntary hospitals and kindred societies throughout the country.

### Chichester Infirmary.

Lieut.-Col. Garnham of Chichester, one of the governors, is apparently very angry with the committee of this institution. He charges the committee with grave irregularities in connection with some recent alterations in the infirmary statutes. He states that the honorary medical officers were required by Statute 35 to attend in rotation every Tuesday to receive and examine in-patients. In the case of a temporary absence arrangements had to be made by each of the honorary medical officers for a colleague to attend, for him. In an emergency, when a medical officer was not able to visit his own patients in the infirmary, he had to engage a colleague or the house surgeon to supply his place. Col. Garnham maintains that none of the honorary medical officers attend on Tuesday to receive and examine patients, and that the whole responsibility for the admission of patients is relegated to the house surgeon for the time being. The rules have now been altered so as to provide that two of the honorary medical officers shall in rotation every Tuesday take up the duties of medical officer of the week. Col. Garnham considers that this regulation gives too much latitude to and throws extra work upon the house surgeon. He maintains that "many governors, especially ladies, object to the examination and the power of refusal of patients by the house surgeon, who is presumably younger and less skilled than the honorary medical officers." Col. Garnham's point appears to be that there are too few honorary medical officers, as the patients have greatly increased during the last six years, and that 30 years ago there were three honorary medical officers on each side, when obtainable. At the present time there are only two honorary medical officers attached to the infirmary in active work, and the time has come for an increase in this number. Col. Garnham condemns several other alterations recently made in the statutes, though the reasons for his condemnation are not very clear. He appears however to make one good point by insisting that the date of the annual meeting is now fixed at such a time that the report cannot be got ready to accompany the notice of meeting, and so entails double expense for postage. This abuse Col. Garnham attributes to an alteration recently made in the statutes. Col. Garnham states that in the opinion of the honorary solicitors of the Infirmary, many of the alterations in the statutes are illegal, a view confirmed by counsel. We know nothing of the merits of this dispute but we call attention to it, because, as it stands, it demands, in our judgment, the issue of a clear statement by the committee, or the calling of a special meeting of governors to dispose of the points at issue once and for all. As matters stand the effect of the dispute on the finances of the infirmary cannot be beneficial.

